# Psychosocial and sociodemographic factors associated with gestational blood glucose levels in women attending public hospitals: Results from baseline of MAASTHI cohort

**DOI:** 10.1371/journal.pone.0293414

**Published:** 2023-10-26

**Authors:** Prafulla Shriyan, Srinidhi Koya, Eunice Lobo, Onno CP van Schayck, Giridhara R. Babu

**Affiliations:** 1 Indian Institute of Public Health-Bangalore, Public Health Foundation of India, Bengaluru, India; 2 School of Social Sciences, Humanities, and Law, Teesside University, Middlesbrough, United Kingdom; 3 Care and Public Health Research Institute, Maastricht University, Maastricht, Limburg, The Netherlands; 4 DBT- Wellcome Trust- India Alliance Senior Research Fellow in Public Health, Hyderabad, India; Kasturba Medical College Mangalore, Manipal Academy of Higher Education, INDIA

## Abstract

**Background:**

Understanding psychosocial environment is important for improving maternal and fetal health outcomes during pregnancy. We aimed to identify the association between gestational blood glucose levels and psychosocial and demographic factors in pregnant women.

**Methods:**

In the MAASTHI pregnancy cohort in Bengaluru, we assessed depressive symptoms, and social support using validated scales at baseline in first trimester. A 2-hour 75 g oral glucose tolerance test (OGTT) was administered between 24–36 weeks of gestation. We examined the relation between psychosocial factors assessed at baseline and gestational blood glucose levels in second/third trimester using multivariate linear regression and explored association between serum cortisol and gestational blood glucose levels in subgroup samples.

**Results:**

We found that 9% of pregnant women had depressive symptoms and 14.3% had Gestational Diabetes Mellitus (GDM). Psychosocial factors, including depressive symptoms, have a significant correlation with gestational fasting(β = 0.12, p-value<0.05) and postprandial blood sugar level(β = 0.23, p-value<0.05) and poor social support were found to have a significant association with gestational fasting blood glucose levels(β = 1.45, p-value <0.05) and postprandial blood sugar levels(β = 2.60, p-value <0.05). The sociodemographic factors such as respondent education, occupation, social and economic status were associated with gestational blood sugar after adjusting for potential confounder variables.

**Conclusion:**

Depressive symptoms and poor social support earlier in pregnancy were significantly associated with increased gestational blood glucose levels. Early detection and recognition of modifiable psychosocial risk factors can reduce glucose intolerance during pregnancy. Evaluating the benefits of screening for psychosocial factors and timely management of gestational diabetes mellitus can be helpful in India.

## Introduction

India has nearly 74.2 million adults aged 20 to 79 years, which is expected to rise to 124.9 million by 2045 [1]. Additionally, the Global burden of diseases (GBD) estimates for India, calculated a 262.9% (225·9 to 304·6) increase in DALY count, and 44% (28·6 to 61·8) increase in age-standardized DALY rate from 1990 to 2021 [2]. Concurrently, the prevalence of Gestational diabetes mellitus (GDM) is also increasing. There is overwhelming evidence suggesting an association between maternal glucose levels and adverse neonatal outcomes [3, 4]. With the increasing prevalence of GDM, it is necessary to understand and act upon the determinants of GDM. Given that India is undergoing a rapid demographical and epidemiological transition, efforts to understand and address modifiable risk factors are imperative. Although considerable evidence is assimilated regarding biological risk factors such as overweight-obesity [5–7], limited information is available regarding the role of contextual psychosocial factors. GDM is associated with several sociodemographic factors. Among these, older age in pregnancy [8–10], family history of diabetes mellitus [9, 11, 12], socioeconomic status, education level, and religion have a role in increasing plasma glucose levels during pregnancy [8, 9, 11].

Studies linking stress and depressive symptoms to increased gestational hyperglycemia are mostly available from developed settings such as Switzerland [13] and the US [14]. In the United States, ethnic minorities, including Hispanic women, have been found to report a higher risk of experiencing elevated stress levels, which is associated with an increased likelihood of glucose intolerance [15]. It is hypothesized that cortisol, the stress hormone mediates the association of psychological factors with diabetes risk. The secretion of cortisol hormone during the poor psychosocial status reduces insulin sensitivity and decreases insulin secretion by acting on pancreatic β-cells [16], hence altering glucose metabolism. Also, social support available to pregnant women, such as maintaining a balanced diet and exercising [17], might be a moderator in this association. Greater social support can also reduce the impact of stress and, thus, reduces the risk of health complications [18, 19]. In support of this, studies in Ireland and Japan demonstrate inverse relation between social support and GDM [20, 21].

The need to address the temporal ambiguity in previously mentioned studies that suggest a possible role for psychosocial and demographic factors in influencing blood plasma glucose levels is the impetus for our research. Cross-sectional and case-control designs have been used primarily in the preceding studies, making it difficult to determine the temporal nature of these associations. To fill this gap in information, our study aims to explore the association between sociodemographic and psychosocial determinants and blood plasma glucose levels during pregnancy. Specifically, our research focuses on examining the psychosocial and demographic factors associated with gestational blood glucose levels in pregnant women visiting public hospitals in urban Bengaluru. We are particularly interested in understanding the influence of two key determinants: depressive symptoms and social support on blood glucose levels in urban Indian women during pregnancy. This will help elucidate the potential mechanisms through which these factors may impact gestational blood glucose levels in urban Indian women. Our hypothesis is that there is an association between depressive symptoms and social support with gestational blood glucose levels in this population. By investigating these associations, we aim to highlight the importance of considering psychosocial and demographic factors in the management and prevention of gestational diabetes in urban settings.

## Materials and methods

**Fig 1:**
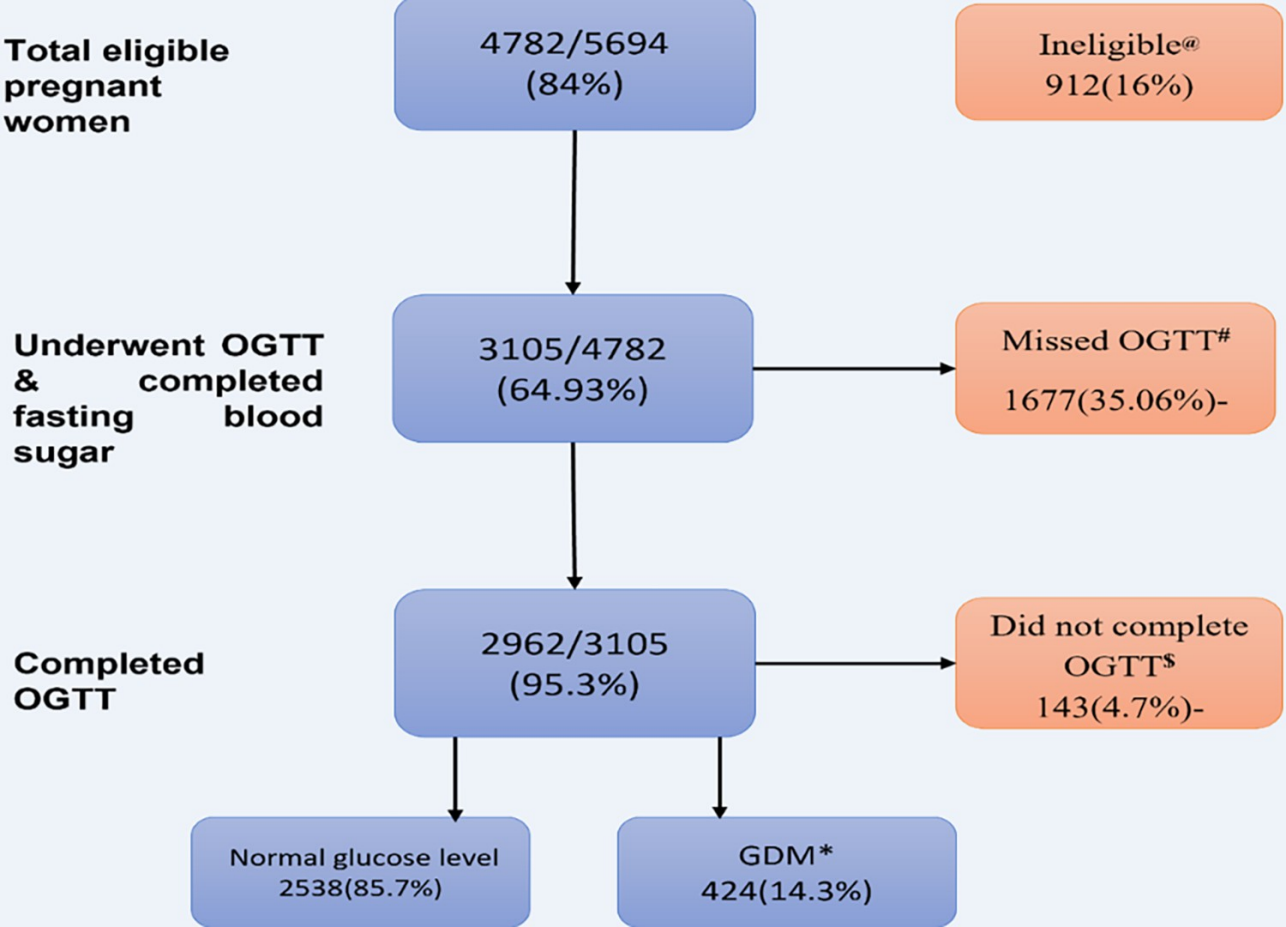
Flow chart representing the number of women approached, screened for eligibility, and considered for the analysis.

We conducted the study from the baseline profile of a large prospective cohort titled “Maternal antecedents of adiposity and studying the transgenerational role of hyperglycemia and insulin(MAASTHI). Details of the cohort have been published earlier [22, 23]. In brief, a cohort of pregnant women was recruited from public hospitals in Urban Bengaluru, a metropolitan city in South India, from 2016 to 2019. In the MAASTHI cohort [3, 24], pregnant women between 18–45 years old, ≥14 weeks of gestation, and visiting a public hospital were approached to participate in the study. After obtaining written informed consent, we recruited the women only if they planned to deliver in the study hospital and were available for future follow-ups in Bengaluru. Exclusion criteria included a history of major co-existing diseases such as diabetes, Human Immunodeficiency Virus (HIV), and Hepatitis B. Oral glucose tolerance test (OGTT) was conducted during 24 to 36 weeks of gestation. The flow of study participants, including screening for eligibility and recruitment, were detailed in Fig 1.

**Fig 2:**
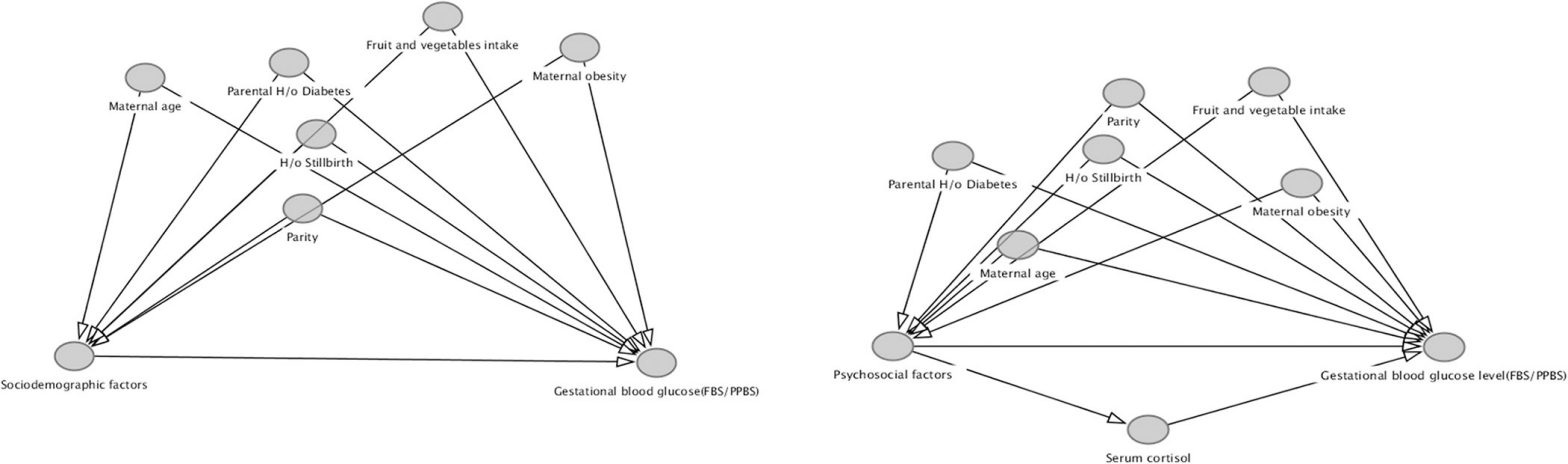
Directed acyclic graph showing study hypothesis with potential confounders.

The directed acyclic graph (Fig 2) showing the association between sociodemographic and psychosocial factors and its association with gestational blood glucose levels with potential confounders.

### Exposure variables

While recruiting at the baseline, we collected information on sociodemographic details, such as age, education, religion, occupation, socioeconomic status, and use of tobacco and alcohol, using standardized questionnaires. As per Kuppuswamy’s Socioeconomic Scale 2017, socioeconomic status was categorized as lower, middle, and upper classes. Psychosocial status was assessed using the Edinburgh Postnatal Depression Scale (EPDS) and social support scale questionnaires validated in Karnataka. This scale has ten questions to assess depressive symptoms. Each question has four possible responses. We asked pregnant women to identify and select the closest response regarding their feelings over the past week. We maintained privacy during questionnaire administration and confidentiality of the data collected. The total score for EPDS ranges from 0 to 30. Based on our previous study in Karnataka [25], a cut-off score of 13 was used for EPDS scores to defining symptoms suggestive of depression. Social support was measured using a 12- item validated questionnaire; items were categorized into good (≥24) and poor (<24) based on a cut-off score of 24 and above [26].

### Outcome variable

Between 24 to 36 weeks, all women were invited to undergo a blood hemoglobin and 2-hour 75 grams OGTT; fasting and postprandial plasma samples were drawn to measure blood glucose levels. Pregnant women were informed to visit the study center in a fasting state. Trained phlebotomists from an accredited laboratory collected venous blood samples to assess fasting blood sugar (FBS). Afterward, 75 grams of anhydrous glucose in 200–300 ml of water was provided to pregnant women. Subsequently, blood for 2-hour postprandial sugar (PPBS) was collected from the pregnant women after 2 hours of taking the glucose solution. Within 30 minutes of blood collection, plasma samples were dispensed to aliquots and delivered to the laboratory in cold boxes for analysis and remaining samples were stored in Biorepository for further analysis. The institution had an MoU with a testing facility that is NABL-accredited used for OGTT and Hb testing. The anemia was classified as normal (blood Hb >11gm/dl), mild (blood Hb = 10.0–10.9gm/dl), and moderate (blood Hb = 7–9.9gm/dl) [27]. GDM was classified following the diagnostic criteria of the World Health Organization (FBS ≥92 mg/dl or PPBS ≥153 mg/dl) [28].

In a sub-sample of stored samples from pregnant women, we estimated the serum cortisol levels. The method employed was an electrochemiluminescence immune assay, and the results were reported in ug/L. We have chosen this method as it is a highly sensitive and specific method of cortisol estimation, which allows for accurate measurement of cortisol levels in a small sample volume. Additionally, the use of an external, National Accreditation Board for testing and calibration laboratories (NABL) accredited laboratory ensures that our results are reliable and of good quality.

### Confounders

Confounders were selected based on a review of existing literature. Clinical factors such as a family history of diabetes [29] previous history of stillbirth are found to be associated with gestational diabetes [30]. maternal characteristics such as smoking status [31], maternal overweight, and obesity [32, 33] and parity [12] were found to be associated with gestational diabetes mellitus [9]. The nutritional practices(low intake of fruits and vegetables) during pregnancy are also significantly associated with gestational diabetes [34].

Obstetric details such as age, gravida, parity, family history of diabetes, and hypertension were collected during the baseline interview. Blood pressure, anthropometry, skinfold thickness, and blood haemoglobin were measured in all women. Hypertension was classified based on the Joint National Committee 8 (JNC8) criteria [prehypertension (SBP 120–139 mmHg OR DBP 80–89 mmHg, hypertension (≥140/90 mmHg)]. The Holtain Calliper (Holtain, UK) was used to assess the skinfold thickness in the biceps, triceps, and subscapular regions. Pregnant women’s total skinfold thickness has been considered an obesity marker rather than BMI. The dietary practices of the pregnant women were obtained through a 24-hr food recall among the consumption of fruits, and vegetables were taken into consideration.

### Quality control

Research assistants were trained and certified in anthropometric measurements by St. Johns Research Institute, Bengaluru. Strict protocols were followed to maintain accuracy. Additionally, inter-and intra-observer reliability of measurements was assessed at the outset. This was followed by annual training and certification by the same institute. Trained phlebotomists collected venous blood for laboratory investigations. Biochemical assays were conducted with internal and external quality checks (QC) at a nationally accredited central laboratory. Internal QC for hemoglobin and plasma glucose was performed by using BIORAD Quality Control samples. The anthropometry equipment was calibrated every month, with the research staff maintaining a calibration log.

### Data management and statistical analysis

Data was collected on Android tablets on the MAASTHI application (app), specially developed for data collection with validation checks. Data were checked periodically for data entry errors and missing information by senior team members. The statistical analysis was performed using IBM SPSS Statistics version 23. In order to explore the psychosocial factors and its association with gestational blood sugar levels, we have done additional subgroup analysis among 230 women with whom we measured serum cortisol levels. The linear regression model was adjusted for potential confounders to estimate adjusted β estimates. We have reported β estimates, 95% confidence interval, and p-values, where p-values <0.05 were considered statistically significant.

## Results

At baseline, 4782 of the 5694 screened pregnant women (84%) were recruited as per the eligibility criteria. Among them, two-thirds of the pregnant women underwent OGTT (n = 3105) between 24 to 36 gestational weeks. The completion rate among those appearing for OGTT was 95.3% (n = 2962); 4.7% could not complete the OGTT because of vomiting and not coming on an empty stomach. We noted that 85.7% of the women had normal glucose levels, and 14.3% were diagnosed with GDM (Fig 1).

The mean age of the participants was 24.26 ± 4.04 years. Most pregnant women and their spouses were high school educated (44% Vs. 39.9%). More than 90% of women (93.2%) were homemakers, while almost half of the spouses were unskilled workers (48.9%). More than half of the women belonged to the lower class (56.8%), and nearly one-third belonged to the middle class (32.10%).

Nearly 20% of the women had one parent with diabetes, and 2.7% had both parents with diabetes. We found that 14.3% of the women had gestational diabetes mellitus (n = 424 of 2962), with mean FBS and PPBS values of 81.85 ± 11.57 mg/dl and 109.05 ± 26.92 mg/dl, respectively. The mean gestational age at OGTT was 28.15 ± 3.12 weeks. Among the recruited participants, 44.5% of the women were anemic. The mean EPDS score in the cohort was 4.40 ± 5.08. We found that 9% of women had symptoms suggestive of depression during pregnancy. Almost three-fourths of the women (73.4%) had a good social support score of ≥ 24 with a mean score of 23.21 ± 10.40 (Table 1).

**Table 1:** Baseline characteristics of eligible pregnant mothers in the MAASTHI birth cohort, Bengaluru, 2016–2019 (n = 3105).

Baseline maternal and pregnancy characteristics	Categories	n (%) / Mean ± SD
**Maternal age (in years)**	Mean	24.26 ± 4.04
	18–25	2012 (65.4)
	26–35	1040 (33.5)
	≥ 36	33 (1.1)
**Gestational age (in weeks)**	Mean	23.94 ± 5.58
**Religion**	Hinduism	1550 (50.1)
	Islam	1428 (46.1)
	Christianity	113 (3.7)
	Others	4 (0.1)
**Participant’s education**	Illiterate	106(3.4)
	Primary and middle school	608(19.6)
	High school	1365(44.1)
	PUC	681(22.0)
	Graduation and above	335(10.8)
**Husband’s education**	Illiterate	285(9.2)
	Primary and middle school	798(25.8)
	High school	1233(39.9)
	PUC	514(16.6)
	Graduation and above	259(8.4)
**Participant’s occupation**	Homemakers	2881 (93.1)
	Unskilled worker	119 (3.8)
	Semi skilled	41(1.3)
	Skilled workers	54(1.7)
**Husband’s occupation**	Unemployed	13 (0.4)
	Unskilled worker	1513(48.9)
	Semi-skilled worker	935(30.2)
	Skilled workers	634(20.5)
**Socioeconomic status (Kuppuswamy Classification)**	Lower class	1757 (56.8)
	Middle class	990 (32.0)
	Upper class	348 (11.2)
**Gravida**	Primi	1209(39.0)
	Multi	1889(61.0)
**Parity**	Nulliparous	1384 (44.7)
	Primipara	1402(45.3)
	Multiparous	312(10.0)
**Abortion**	Yes	604(19.4)
**Stillbirth**	Yes	53 (1.7)
**Family history of diabetics**	None	2380(77.3)
	One parent	614(19.9)
	Both parents	84 (2.7)
**Tobacco smoking by participant**	Yes	4 (0.2)
**Smokeless tobacco consumption by participant**	Yes	13 (0.5)
**Alcohol consumption by the participant**	Yes	3 (0.1)
**Tobacco smoking by husband**	Yes	955 (31.3)
**Smokeless tobacco consumption by husband**	Yes	194 (6.6)
**Alcohol consumption by husband**	Yes	424 (13.8)
**EPDS Score**	Mean±SD	4.40 ± 5.08
	13 or above)	279 (9.1)
**Social Support Score**	Mean ±SD	23.21 ± 10.40
	24 or above	2262 (73.4)
**Systolic Blood Pressure (mmHg)**	Mean±SD	102.86 ± 10.88
**Diastolic Blood Pressure (mmHg)**	Mean±SD	64.92 ± 9.02
**Hypertension**	No	2713 (92.3)
	Prehypertensive	204 (6.9)
	Hypertensive	11 (0.4)
**Haemoglobin (gm/dl)**	Mean±SD	10.9± 1.26
**Anaemia**	Yes	1374 (44.5)
**Fasting Blood Sugar (mg/dl)**	Mean±SD	81.85 ± 11.57
**Postprandial Blood Sugar (mg/dl)**	Mean ±SD	109.05 ± 26.92
**Gestational Diabetes Mellitus during the current pregnancy**	Yes	424 (14.3)
**Gestational age at the time of OGTT (in weeks)**	Mean ±SD	28.15 ± 3.12
**Vegetable consumption**	Daily	1995(69.2)
	Weekly	826(28.7)
	Rarely	60(2.1)
**Fruit consumption**	Daily	1896(65.8)
	Weekly	914(31.7)
	Rarely	71(2.5)

PUC: Pre-University College, GDM: FBS ≥92 mg/dl or PPBS ≥153 mg/dl; Anemia: Haemoglobin <11g/dl, Hypertension: prehypertension (SBP 120–139 mmHg OR DBP 80–89 mmHg, hypertension (≥140/90 mmHg), EPDS-Edinburgh Postnatal Depression Score

**Fig 3:**
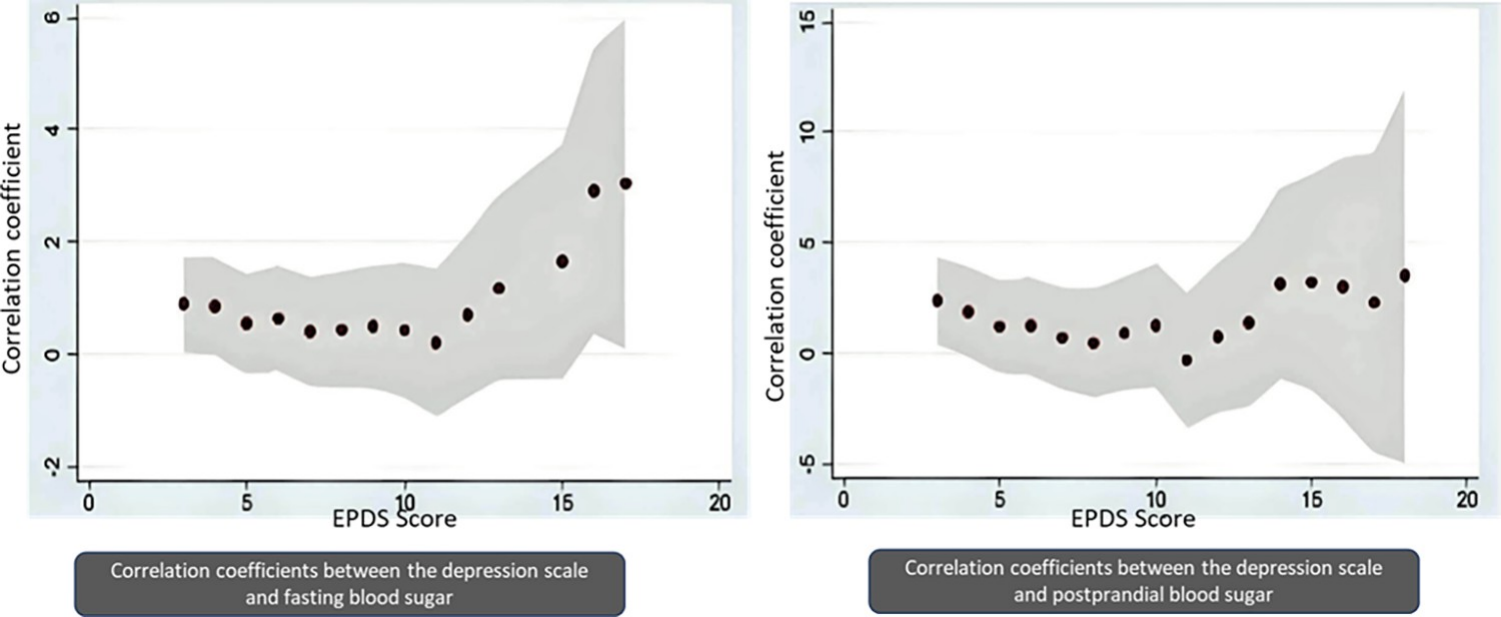
Area graph showing trend in correlation coefficients observed between EPDS score and fasting, postprandial blood sugar. EPDS- Edinburgh Postnatal Depression Score, FBS-fasting blood sugar, PPBS- postprandial blood sugar.

The area graph using the correlation coefficients obtained through multivariate linear regression is presented in Fig 3. The EPDS score cut-off as an independent variable and fasting and postprandial blood sugar levels as dependent variables; there is a trend of increasing correlation coefficients with fluctuations are seen with an increase in the EPDS cut-off score for both fasting and postprandial blood sugar (S1 File).

Tables 2 and 3 summarize the sociodemographic and psychosocial factors affecting FBS levels and PPBS levels, respectively.

**Table 2:** Sociodemographic and psychosocial factors affecting FBS levels of pregnant women in the MAASTHI birth cohort, Bengaluru, 2016-2019(n = 3105).

Factors	Unadjusted			Adjusted		
	β Estimate	p-value	95% CI	β Estimate	p-value	095% CI
**Respondent education**						
Illiterate	-.119	0.92	(-2.25, 2.80)	-.333	0.79	(-2.86, 2.20)
Primary and middle school	-.911	0.43	(-3.34, 1.39)	-.963	0.46	(-3.40,1.47)
High school	-.972	0.42	(-3.19, 1.37)	-1.08	0.40	(-3.61, 1.440
PUC	0.272	0.83	(-2.50, 2.26)	-.545	0.69	(-3.27, 2.19)
Graduation and above(Ref)	**1**					
**Husband Education**						
Illiterate	0.453	0.57	(-1.11, 2.02)	-.824	0.44	(-2.94, 1.29)
Primary and middle school	0.445	0.55	(-1.04,1.93)	.882	0.34	(-.92, 2.69)
High school	0.809	0.34	(-.86, 2.48)	.577	0.48	(-1.02, 2.18)
PUC	-.280	0.77	(-2.22, 1.67)	.428	0.61	(-1.24, 2.10)
Graduation and above (Ref)	1					
**Res. occupation**						
Homemakers	1.062	0.32	(-1.06, 3.18)	1.389	0.21	(-.81, 3.59)
Unskilled worker	**4.587**	**0.01**	**(1.02, 8.15)**	**3.830**	**0.05**	**(-0.02, 7.68)**
Semi skilled	-.077	0.96	(-3.19, 3.03)	.264	0.88	(-3.15, 3.68)
Skilled workers(Ref)	**1**					
**Hus. occupation**						
Unemployed	1.948	0.54	(-4.37, 8.27)	-.502	0.90	(-8.50,7.53)
Unskilled worker	1.740	0.59	(-4.59, 8.08)	-.556	0.89	(-8.61, 7.50)
Semi skilled	2.137	0.51	(-4.22, 8.49)	-.671	0.87	(-8.75, 7.40)
Skilled workers(Ref)	1			**1**		
**Socio economic status**						
Lower class	-.217	0.63	(-1.11, 0.68)	-.106	0.83	(-1.07, 0.86)
Middle class	-.567	0.40	(-1.89, 0.76)	**-1.367**	**0.05**	**(-2.78, 0.05)**
Upper class(Ref)						
**Religion**						
Islam	1.442	0.80	(-10.05,12.93)	-.856	0.89	(-13.91, 12.19)
Christian	0.983	0.86	(-10.32,12.29)	1.076	0.87	(-11.98, 14.13)
Others	-1.286	0.82	(-12.59,10.02)	1.583	0.815	(-11.66, 14.82)
Hindu(Ref)	1			1		
**Husband cigarette**						
Yes	4.241	0.30	(-3.81, 12.29)	3.140	0.47	(-5.47, 11.75)
No	**1**			**1**		
**Abortion**						
Yes	**2.317**	**0.00**	**(1.28, 3.34)**	**1.927**	**0.001**	**(.82, 3.03)**
No(ref)	**1**			**1**		
**EPDS score**	**0.105**	**0.01**	**(0.02, 0.18)**	**0.125**	**0.004**	**(0.03, 0.21)**
**Poor support (<24)**	**1.323**	**0.00**	**(.39, 2.24)**	**1.45**	**0.004**	**(0.46, 2.44)**
**Good support (24 and above)**	**1**			**1**		

Model is adjusted for set of maternal factors such as age, parental history of diabetes, history of still birth, parity, skinfold thickness above the 90^th^ percentile (obesity marker), and dietary practices such as fruit intake and vegetable intake.

**Table 3:** Sociodemographic and psychosocial factors affecting PPBS levels of pregnant women in the MAASTHI birth cohort, Bengaluru, 2016–2019 (n = 2962).

	Unadjusted			Adjusted		
Factors	β Estimate	p-value	95% CI	β Estimate	p-value	095% CI
**Respondent Education**						
Illiterate	-4.021	0.16	(-9.75, 1.70)	**-6.188**	**0.04**	**(-12.12, -0.25)**
Primary and middle school	-3.448	0.21	(-8.92, 2.02)	-4.482	0.12	(-10.17, 1.21)
High school	-3.475	0.23	(-9.15, 2.20)	-4.902	0.10	(-10.82, 1.02)
PUC	1.757	0.56	(-4.27, 7.79)	-3.501	0.28	(-9.86, 2.86)
Graduation and above	**1**			**1**		
**Hus. education**						
Illiterate	0.077	0.96	(-3.72, 3.87)	0.462	0.81	(-3.45, 4.41)
Primary and middle school	-.192	0.91	(-3.81, 3.42)	-.618	0.75	(-4.42, 3.18)
High school	1.132	0.58	(-2.92, 5.19)	0.327	0.88	(-3.95, 4.60)
PUC	1.475	0.53	(-3.19, 6.14)	-1.718	0.49	(-6.69, 3.25)
Graduation and above	1			**1**		
**Res. occupation**						
Homemakers	4.286	0.11	(-.98, 9.55)	4.449	0.14	(-.91,9.81)
Unskilled worker	**9.687**	**0.02**	**(1.17, 18.19)**	6.576	0.15	(-2.45,15.60)
Semi skilled	2.440	0.52	(-5.16,10.04)	1.245	0.76	(-6.98, 9.47)
Skilled workers	**1**			**1**		
**Hus. occupation**						
H. Unemployed	-2.727	0.72	(-17.99,12.54)	-11.35	0.22	(-29.57, 6.87)
Unskilled worker	-3.307	0.67	(-18.61, 12.0)	-11.82	0.20	(-30.08, 6.43)
Semi skilled	1.821	0.81	(-13.53,17.18)	-8.229	0.37	(-26.54, 10.08)
Skilled worker	1			**1**		
**Socio economic status**						
Lower class	-.514	0.75	(-3.71, 2.68)	.180	0.87	(-2.11, 2.42)
Middle class	0.948	0.39	(-1.24, 3.13)	**-3.392**	**0.04**	**(-6.71, -.06)**
Upper class (Ref)	**1**					
**Religion**						
Islam	1.442	0.80	(-10.05,12.93)	-.302	0.98	(-30.05, 29.44)
Christian	0.983	0.86	(-10.32,12.29)	-.665	0.96	(-30.41, 29.08)
Others	-1.286	0.82	(-12.59,10.02)	-.193	0.99	(-30.41, 30.03)
Hindu	1			1		
**Husband cigarette**						
Yes	-.706	0.94	(-20.50,19.09)	2.53	0.81	(-18.18, 23.25)
No	**1**			**1**		
**Abortion**						
Yes	**6.02**	**0.000**	**(3.54, 8.54)**	**5.41**	**0.000**	**(2.79, 8.02)**
No	**1**			**1**		
**EPDS score**	0.17	0.08	(-.02, 0.36)	**0.23**	**0.02**	**(0.03, 0.43)**
Poor support (<24)	**2.312**	**0.04**	**(.06, 4.56)**	**2.60**	**0.02**	**(0.26, 4.94)**
Good support (24 and above)	**1**					

Model is adjusted for set of maternal factors such as age, family history of diabetes, previous history of stillbirth, parity, skinfold thickness above the 90^th^ percentile, and dietary practices such as fruit intake and vegetable intake.

After adjusting for potential confounders, we found that FBS levels were higher by 3.83 units among women who were working as unskilled workers compared to women who were skilled workers (p-value = 0.05). When compared to women who belonged to the upper socioeconomic class, middle-class women were found to have lower FBS by 1.36 units (p-value = 0.05). Women with a previous history of abortion were found to have FBS levels higher by 1.92 units when compared to those who do not have an abortion history (P-value = 0.001).

Further, compared to women who graduated and above, PPBS levels were lower by 6.18 units (p-value = 0.04) among women who were illiterate. PPBS levels were higher by 9.68 units (p-value = 0.02) among women working as unskilled workers compared to women who were skilled workers. After adjusting for the effect of potential confounders(age, family history of diabetes, H/o stillbirth, parity, skinfold thickness above the 90^th^ percentile, and fruit intake and vegetable intake), significant associations were not observed. Compared to upper-class women, women who belonged to the middle socioeconomic class were found to have lower PPBS levels by 3.39 units (p-value = 0.04). Women with previous H/o abortion were found to have PPBS levels higher by 5.41 units (p-value = 0.000) when compared to those women who do not have H/o abortion.

Among psychosocial factors, depression scores and poor social support during pregnancy were significantly associated with fasting and postprandial blood sugar levels. For every unit increase in depression scores during pregnancy, the FBS levels increase by 0.12 units (p-value = 0.004), whereas PPBS levels increase by 0.23 units (p-value = 0.02). For women with poor social support, we noted that FBS levels were higher by 1.45 units (p-value = 0.004), whereas PPBS levels were higher by 2.60 units (p-value: 0.02) when compared to women with social support scores≧24. Both unadjusted and adjusted estimates for depression scores and poor social support scores were found to be statistically significant with FBS and PPBS levels (Tables 2 and 3).

There was no significant association between blood glucose levels observed for religion, spousal smoking status, husband’s education, and occupation.

Women with serum cortisol levels higher than the mean have been found to have FBS levels higher by 1.19 units and PPBS levels higher by 2.92 units. The results are not statistically significant (Table 4).

**Table 4:** Depression biomarker serum cortisol and its association with gestational blood glucose level(n = 230).

Variable	Unadjusted			Adjusted		
	Fasting blood sugar					
	B	p-value	95%CI	B	p-value	95%CI
S. cortisol >mean cut-off (≥17.66ug/L)	1.433	0.22	(-.89, 3.76)	1.191	0.30	(-1.07, 3.46)
S. cortisol <mean cut-off	1			1		
	**Postprandial blood sugar**					
S. cortisol >mean cut-off (≥17.66ug/L)	4.507	0.20	(-2.46, 11.48)	2.927	0.38	(-3.68, 9.54)
S. cortisol <mean cut-off	1		.	.1		

Model adjusted for maternal age, parity, parental history of DM, total skinfold thickness >90^th^ percentile, and intake of fruits and vegetables during pregnancy.

## Discussion

In a prospective study in urban India, we found that 14.3% of the women had GDM, and 9% had symptoms suggestive of depression during pregnancy. We found that gestational blood glucose levels were strongly and inversely associated with social support and directly with higher depression scores. We also found that sociodemographic factors such as education, occupation of the respondent, and socioeconomic status were associated with gestational blood glucose levels. Sub group analysis on stress biomarker serum cortisol also showed positive association with gestational blood glucose levels.

Our findings on the relationship between depressive symptoms and blood sugar levels are consistent with the existing literature. A meta-analysis report suggests that stress during pregnancy increases the incidence of gestational diabetes mellitus [35]. Horsch A et al. found that women exposed to stress during pregnancy were associated with higher fasting blood sugar concentrations [13]. The study by Byrn M et al. found that depression during pregnancy was a risk factor for the development of GDM [36]. This is consistent with the results of Egan A M et al. and Morrison C et al., who showed that women with GDM had elevated depression scores [37, 38]. Depression and diabetes have a bi-directional relationship. Depression is seen in people with diabetes and is commonly believed to be linked to this chronic disease. People who are depressed are either obese or have insulin resistance. Inflammatory cytokines could impact the metabolic pathway and could even be attributed to the disease in the future [39, 40]. Therefore, screening and offering support services to pregnant women with depression is important in reducing the propensity of gestational hyperglycemia [41].

Our results are consistent with the existing knowledge that poor social support significantly increases blood glucose levels during pregnancy. A study conducted in Ireland found that women having GDM reported lower levels of social support [20, 42]. Similarly, a systematic review conducted in 2014 and a quantitative study conducted by Ruggiero et al. found that social support was a key factor in GDM management [43, 44]. Women’s capacity to change their lifestyles and adhere to medical advice to ensure the best possible maternal and fetal health depends on the level of social support they receive from their partners and family [45]. Hospitals must consider counselling the pregnant woman’s family to provide sufficient social support during pregnancy. In general, an awareness about providing appropriate emotional and social support for pregnant women could positively benefit the women. Proper medical support in terms of information and recommendation of preventive practices for GDM to pregnant women will reduce the future risk of developing GDM.

Our findings provide important socio-demographic health information at an initial pregnancy visit to identify pregnant women who might be at higher risk for gestational hyperglycaemia. First, as reported in this study, unskilled workers and their association with higher gestational blood sugar levels are well documented [46]. Second, socioeconomic status and education level were found to be significant risk factors. The existing evidence from China shows that high socio economic status was associated with a reduced risk of GDM [47]. Whereas studies from India support our finding that high socioeconomic status and high education are associated with increased odds of GDM [48]. During screening for gestational diabetes mellitus, government hospitals should consider including the woman’s education, occupation, and socioeconomic status factors. GDM screening practices are quite poor in government hospitals [49]. Including more detailed assessments on the psychosocial and demographic profile is a cost-effective way of initially screening GDM mothers.

Our findings cannot confirm the plausible association between elevated serum cortisol levels and increased fasting blood sugar (FBS) and postprandial blood sugar (PPBS) levels in women. Our results were not statistically significant; further research with larger sample sizes may be necessary to delineate the association. Since cortisol level fluctuates throughout the day, future studies can aim at early morning measurement of free cortisol levels and while controlling for other factors that may affect cortisol levels such as stress, sleep, and physical activity.

### Strength and limitations

Our study was conducted in the public health facilities used by most pregnant women for their antenatal and postnatal check-ups. We used validated study tools in India to assess our study variables. Quality check parameters were applied in the study to obtain accurate and reliable findings. The generalizability of the results to rural areas is limited since it was conducted in urban public health facilities. Another limitation of the study is that one-third of women did not appear for or complete the OGTT, citing reasons such as disinterest, disagreement by the in-laws or husbands, and a change in residence. The reasons for not completing or appearing for the OGTT tests reflect a lack of social awareness. Therefore, probability of selection bias is limited, as loss to follow-up is not related to outcome (GDM). Also, the non-significant association between serum cortisol and gestational blood glucose levels needs to be examined further. Despite the study’s limitations, the findings contributed to identifying important causes of elevated gestational blood glucose levels in pregnant women.

## Public health implications

The study’s findings shed light on potential sociodemographic and psychosocial risk factors linked to rising blood sugar levels during pregnancy. This will emphasise emphasize the importance of a more thorough investigation, which might make use of retrospective cohort studies. The assessment of modifiable risk variables is a useful diagnostic tool for identifying women who are at risk for developing gestational glucose intolerance. The pregnant women who receive care at the facility are going to be benefitted. This could be a crucial step in reducing the negative effects of gestational glucose intolerance, which are on the rise due to global increases in obesity levels and the number of older pregnancies.

## Conclusion

Early detection of modifiable risk factors, such as poor social support and depressive symptoms, in urban populations can reduce the burden of gestational glucose intolerance. Using a risk profile incorporating both socio-demographic and psychosocial factors can assist in prioritizing screening and management for GDM. Healthcare providers should be educated on the value of early GDM detection and expectant mothers, and families sensitized to GDM risk factors to ensure timely management.

## Supporting information

S1 File Regression analysis showing a correlation between different EPDS score cut-offs and fasting and postprandial blood sugar levels. (PDF)

## Abbreviations

MAASTHI: Maternal antecedents of adiposity and studying the transgenerational role of hyperglycemia and insulin
EPDS: Edinburgh Postnatal Depression Scale
OGTT: Oral Glucose Tolerance Test
GDM: Gestational Diabetes Mellitus
T2DM: Type 2 Diabetes Mellitus
LMIC: Low and Middle-Income Countries
HIC: High-Income Countries
FBS: Fasting Blood Sugar
PPBS: Postprandial Blood Sugar
