# Correction: Psychosocial and sociodemographic factors associated with gestational blood glucose levels in women attending public hospitals: Results from baseline of MAASTHI cohort

**DOI:** 10.1371/journal.pone.0317157

**Published:** 2025-01-02

**Authors:** Prafulla Shriyan, Srinidhi Koya, Eunice Lobo, Onno CP van Schayck, Giridhara R. Babu

The affiliation for the fifth author is incorrect. The correct affiliation is not indicated. Giridhara R. Babu is not affiliated with #1 and #4 but with: Department of Population Medicine, College of Medicine, QU Health, Qatar University.
