# Supplementary material for: Psychosocial and sociodemographic factors associated with gestational blood glucose levels in women attending public hospitals: Results from baseline of MAASTHI cohort
Source: PLoS One. 2023 Oct 26;18(10):e0293414. doi: 10.1371/journal.pone.0293414 (PMC10602325; doi:10.1371/journal.pone.0293414)
Supplement: S1 File — (PDF) [file pone.0293414.s001.pdf]

S1 File. Regression analysis showing a correlation between different EPDS score cut-offs and fasting and postprandial blood sugar levels

Table 1-15. Linear regression analysis showing EPDS cut-off score of 3 and above and its correlation with fasting blood sugar level.

Table 1. EPDS cut off score 3 and its correlation with fasting blood sugar

Baseline_prifbs1	Coefficient	Std. err.	t	P>|t|	[95% conf.	interval]
epds3	.9032951	.4277764	2.11	0.035	.0645166	1.742073
Baseline_psoscore	-.0109653	.0201074	-0.55	0.586	-.0503916	.028461
Baseline_page	.4423541	.0573756	7.71	0.000	.3298526	.5548555
Baseline_redu	-.0140651	.202793	-0.07	0.945	-.4116989	.3835688
Baseline_hedu	.2364184	.1949521	1.21	0.225	-.1458412	.6186781
Baseline_pses	-.4779697	.3695061	-1.29	0.196	-1.202493	.2465532
Baseline_reli	1.069746	.225855	4.74	0.000	.6268928	1.5126
Baseline_par	-.4474539	.3509561	-1.27	0.202	-1.135604	.2406962
Baseline_fdm	2.212602	.6644372	3.33	0.001	.9097825	3.515422
Baseline_mdm	2.592166	.5994732	4.32	0.000	1.416727	3.767605
_cons	69.92553	1.582174	44.20	0.000	66.82322	73.02784

Table 2.EPDS cut off score 4 and its correlation with fasting blood sugar

Baseline_prifbs1	Coefficient	Std. err.	t	P>|t|	[95% conf.	interval]
epds4	.8634631	.4363933	1.98	0.048	.0077886	1.719138
Baseline_psoscore	-.0109711	.0201225	-0.55	0.586	-.0504272	.0284849
Baseline_page	.4436136	.0573736	7.73	0.000	.3311161	.556111
Baseline_redu	-.0137054	.2028127	-0.07	0.946	-.411378	.3839673
Baseline_hedu	.2338041	.1950017	1.20	0.231	-.1485527	.6161609
Baseline_pses	-.489831	.3692286	-1.33	0.185	-1.21381	.2341476
Baseline_reli	1.072951	.2258353	4.75	0.000	.6301359	1.515766
Baseline_par	-.4492766	.3509812	-1.28	0.201	-1.137476	.2389228
Baseline_fdm	2.266464	.6649486	3.41	0.001	.962641	3.570286
Baseline_mdm	2.571776	.5994622	4.29	0.000	1.396358	3.747194
_cons	69.9811	1.580556	44.28	0.000	66.88196	73.08023

Table 3.EPDS cut off score 5 and its correlation with fasting blood sugar

Baseline_prifbs1	Coefficient	Std. err.	t	P>|t|	[95% conf.	interval]
epds5	.5538903	.4482624	1.24	0.217	-.325057	1.432838
Baseline_psoscore	-.0125905	.0201268	-0.63	0.532	-.0520549	.0268739
Baseline_page	.4442103	.0573961	7.74	0.000	.3316688	.5567518
Baseline_redu	-.0158431	.2028961	-0.08	0.938	-.4136792	.381993
Baseline_hedu	.2340008	.1951659	1.20	0.231	-.1486779	.6166796
Baseline_pses	-.5064006	.3696548	-1.37	0.171	-1.231215	.2184137
Baseline_reli	1.084374	.2258652	4.80	0.000	.6415003	1.527247
Baseline_par	-.4534175	.3511189	-1.29	0.197	-1.141887	.235052
Baseline_fdm	2.251283	.6653196	3.38	0.001	.9467332	3.555833
Baseline_mdm	2.56508	.5997452	4.28	0.000	1.389108	3.741053
_cons	70.17399	1.578775	44.45	0.000	67.07834	73.26963

Table 4.EPDS cut off score 6 and its correlation with fasting blood sugar

Baseline_prifbs1	Coefficient	Std. err.	t	P>|t|	[95% conf.	interval]
epds6	.6492561	.4727073	1.37	0.170	-.2776222	1.576134
Baseline_psoscore	-.0118978	.0201583	-0.59	0.555	-.0514238	.0276283
Baseline_page	.4442876	.0573922	7.74	0.000	.3317538	.5568215
Baseline_redu	-.0194643	.202907	-0.10	0.924	-.4173218	.3783931
Baseline_hedu	.232058	.1951803	1.19	0.235	-.1506492	.6147651
Baseline_pses	-.4998128	.3697753	-1.35	0.177	-1.224863	.2252379
Baseline_reli	1.08219	.2258702	4.79	0.000	.6393066	1.525073
Baseline_par	-.4566848	.351077	-1.30	0.193	-1.145072	.2317024
Baseline_fdm	2.243559	.6649915	3.37	0.001	.939652	3.547466
Baseline_mdm	2.556776	.5997906	4.26	0.000	1.380714	3.732837
_cons	70.17392	1.575922	44.53	0.000	67.08388	73.26397

Table 5.EPDS cut off score 7 and its correlation with fasting blood sugar

Baseline_prifbs1	Coefficient	Std. err.	t	P>|t|	[95% conf.	interval]
epds7	.4178166	.4937683	0.85	0.398	-.5503579	1.385991
Baseline_psoscore	-.0132115	.0201538	-0.66	0.512	-.0527288	.0263059
Baseline_page	.4446941	.0574038	7.75	0.000	.3321374	.5572507
Baseline_redu	-.0183799	.2029597	-0.09	0.928	-.4163408	.379581
Baseline_hedu	.2368587	.1951946	1.21	0.225	-.1458764	.6195937
Baseline_pses	-.5149653	.3700989	-1.39	0.164	-1.24065	.2107198
Baseline_reli	1.087551	.2260142	4.81	0.000	.6443852	1.530717
Baseline_par	-.4576147	.3511472	-1.30	0.193	-1.14614	.2309102
Baseline_fdm	2.233631	.6651241	3.36	0.001	.9294639	3.537798
Baseline_mdm	2.562815	.5999179	4.27	0.000	1.386503	3.739126
_cons	70.27718	1.575976	44.59	0.000	67.18703	73.36733

Table 6.EPDS cut off score 8 and its correlation with fasting blood sugar

Baseline_prifbs1	Coefficient	Std. err.	t	P>|t|	[95% conf.	interval]
epds8	.4454277	.51882	0.86	0.391	-.5718678	1.462723
Baseline_psoscore	-.0131047	.0201644	-0.65	0.516	-.0526428	.0264334
Baseline_page	.4449706	.0574055	7.75	0.000	.3324107	.5575306
Baseline_redu	-.0183326	.202957	-0.09	0.928	-.416288	.3796228
Baseline_hedu	.2375486	.1951612	1.22	0.224	-.1451211	.6202182
Baseline_pses	-.5180699	.3697218	-1.40	0.161	-1.243016	.2068758
Baseline_reli	1.086365	.2260913	4.80	0.000	.6430477	1.529681
Baseline_par	-.4602205	.3511524	-1.31	0.190	-1.148756	.2283147
Baseline_fdm	2.23421	.6651332	3.36	0.001	.930025	3.538394
Baseline_mdm	2.569388	.5998078	4.28	0.000	1.393292	3.745483
_cons	70.28041	1.5752	44.62	0.000	67.19178	73.36904

Table 7.EPDS cut off score 9 and its correlation with fasting blood sugar

Baseline_prifbs1	Coefficient	Std. err.	t	P>|t|	[95% conf.	interval]
epds9	.4991138	.5556973	0.90	0.369	-.5904904	1.588718
Baseline_psoscore	-.0130827	.0201547	-0.65	0.516	-.0526017	.0264363
Baseline_page	.4454776	.0574121	7.76	0.000	.3329047	.5580506
Baseline_redu	-.0207539	.2030165	-0.10	0.919	-.4188261	.3773183
Baseline_hedu	.2365377	.1951887	1.21	0.226	-.1461858	.6192613
Baseline_pses	-.5165187	.3697459	-1.40	0.163	-1.241512	.2084743
Baseline_reli	1.085018	.2261459	4.80	0.000	.6415944	1.528442
Baseline_par	-.4629289	.3511791	-1.32	0.188	-1.151516	.2256586
Baseline_fdm	2.229441	.6649777	3.35	0.001	.925561	3.53332
Baseline_mdm	2.561959	.5999135	4.27	0.000	1.385656	3.738262
_cons	70.28639	1.573526	44.67	0.000	67.20104	73.37174

Table 8.EPDS cut off score 10 and its correlation with fasting blood sugar

Baseline_prifbs1	Coefficient	Std. err.	t	P>|t|	[95% conf.	interval]
epds10	.4369003	.6070357	0.72	0.472	-.7533676	1.627168
Baseline_psoscore	-.0135809	.0201424	-0.67	0.500	-.0530758	.025914
Baseline_page	.444931	.0574081	7.75	0.000	.332366	.557496
Baseline_redu	-.0178906	.2029665	-0.09	0.930	-.4158648	.3800836
Baseline_hedu	.2374802	.1952188	1.22	0.224	-.1453022	.6202627
Baseline_pses	-.5214422	.3698586	-1.41	0.159	-1.246656	.2037717
Baseline_reli	1.087707	.2261815	4.81	0.000	.6442134	1.531201
Baseline_par	-.4601187	.3511678	-1.31	0.190	-1.148684	.2284466
Baseline_fdm	2.225621	.6649734	3.35	0.001	.9217495	3.529492
Baseline_mdm	2.562084	.6000108	4.27	0.000	1.385591	3.738578
_cons	70.32993	1.571715	44.75	0.000	67.24813	73.41173

Table 9.EPDS cut off score 11 and its correlation with fasting blood sugar

Baseline_prifbs1	Coefficient	Std. err.	t	P>|t|	[95% conf.	interval]
epds11	.2160132	.6637102	0.33	0.745	-1.085381	1.517408
Baseline_psoscore	-.0144524	.0201414	-0.72	0.473	-.0539453	.0250405
Baseline_page	.444446	.0574103	7.74	0.000	.3318766	.5570154
Baseline_redu	-.0155785	.2029496	-0.08	0.939	-.4135194	.3823625
Baseline_hedu	.241427	.1951458	1.24	0.216	-.1412124	.6240665
Baseline_pses	-.5368369	.3693031	-1.45	0.146	-1.260962	.1872879
Baseline_reli	1.094109	.2262429	4.84	0.000	.6504949	1.537723
Baseline_par	-.4586441	.3511854	-1.31	0.192	-1.147244	.2299556
Baseline_fdm	2.22223	.6650684	3.34	0.001	.918173	3.526288
Baseline_mdm	2.568996	.5999915	4.28	0.000	1.39254	3.745452
_cons	70.39786	1.569509	44.85	0.000	67.32039	73.47533

Table 10.EPDS cut off score 12 and its correlation with fasting blood sugar

Baseline_prifbs1	Coefficient	Std. err.	t	P>|t|	[95% conf.	interval]
epds12	.711562	.7367026	0.97	0.334	-.732955	2.156079
Baseline_psoscore	-.0129309	.0201537	-0.64	0.521	-.052448	.0265862
Baseline_page	.4447879	.0574019	7.75	0.000	.3322349	.5573408
Baseline_redu	-.0139365	.2029201	-0.07	0.945	-.4118197	.3839468
Baseline_hedu	.2413005	.1950546	1.24	0.216	-.1411601	.623761
Baseline_pses	-.5279747	.3687883	-1.43	0.152	-1.25109	.1951406
Baseline_reli	1.084252	.2261155	4.80	0.000	.6408872	1.527616
Baseline_par	-.4605808	.3511413	-1.31	0.190	-1.149094	.2279325
Baseline_fdm	2.239298	.6652083	3.37	0.001	.934966	3.54363
Baseline_mdm	2.567576	.5997991	4.28	0.000	1.391497	3.743654
_cons	70.30475	1.570479	44.77	0.000	67.22538	73.38413

Table 11.EPDS cut off score 13 and its correlation with fasting blood sugar

Baseline_prifbs1	Coefficient	Std. err.	t	P>|t|	[95% conf.	interval]
Epds13	1.185097	.8281141	1.43	0.153	-.4386581	2.808853
Baseline_psoscore	-.0127076	.0200906	-0.63	0.527	-.0521009	.0266857
Baseline_page	.445008	.0573912	7.75	0.000	.3324761	.55754
Baseline_redu	-.017103	.2028816	-0.08	0.933	-.4149106	.3807047
Baseline_hedu	.2390155	.1950289	1.23	0.220	-.1433947	.6214257
Baseline_pses	-.5238174	.3685888	-1.42	0.155	-1.246542	.1989068
Baseline_reli	1.077005	.2260612	4.76	0.000	.6337471	1.520263
Baseline_par	-.4643446	.3510909	-1.32	0.186	-1.152759	.2240699
Baseline_fdm	2.246173	.665004	3.38	0.001	.9422421	3.550105
Baseline_mdm	2.565273	.5996808	4.28	0.000	1.389427	3.74112
_cons	70.29489	1.567292	44.85	0.000	67.22176	73.36801

Table 12.EPDS cut off score 15 and its correlation with fasting blood sugar

Baseline_prifbs1	Coefficient	Std. err.	t	P>|t|	[95% conf.	interval]
epds15	1.654265	1.058772	1.56	0.118	-.4217618	3.730292
Baseline_psoscore	-.0125748	.0200842	-0.63	0.531	-.0519558	.0268061
Baseline_page	.4456112	.0573905	7.76	0.000	.3330807	.5581416
Baseline_redu	-.0138851	.2028648	-0.07	0.945	-.4116598	.3838897
Baseline_hedu	.2441165	.1949951	1.25	0.211	-.1382273	.6264603
Baseline_pses	-.5315726	.3683641	-1.44	0.149	-1.253856	.190711
Baseline_reli	1.083825	.2257166	4.80	0.000	.6412429	1.526407
Baseline_par	-.471387	.3511417	-1.34	0.180	-1.159901	.2171272
Baseline_fdm	2.250384	.6649903	3.38	0.001	.9464801	3.554289
Baseline_mdm	2.568226	.5996231	4.28	0.000	1.392492	3.743959
_cons	70.27705	1.56738	44.84	0.000	67.20375	73.35034

Table 13.EPDS cut off score 16 and its correlation with fasting blood sugar

Baseline_prifbs1	Coefficient	Std. err.	t	P>|t|	[95% conf.	interval]
epds16	2.91797	1.28502	2.27	0.023	.3983181	5.437621
Baseline_psoscore	-.0123246	.0200455	-0.61	0.539	-.0516297	.0269804
Baseline_page	.4469741	.0573694	7.79	0.000	.3344849	.5594633
Baseline_redu	-.0194787	.2027768	-0.10	0.923	-.4170808	.3781234
Baseline_hedu	.2487185	.1949178	1.28	0.202	-.1334738	.6309108
Baseline_pses	-.5290502	.3681565	-1.44	0.151	-1.250927	.1928263
Baseline_reli	1.074476	.225655	4.76	0.000	.6320141	1.516937
Baseline_par	-.4745812	.3509498	-1.35	0.176	-1.162719	.2135567
Baseline_fdm	2.260637	.664619	3.40	0.001	.9574605	3.563813
Baseline_mdm	2.56331	.5993485	4.28	0.000	1.388115	3.738505
_cons	70.23755	1.565744	44.86	0.000	67.16746	73.30764

Table 14.EPDS cut off score 17 and its correlation with fasting blood sugar

Baseline_prifbs1	Coefficient	Std. err.	t	P>|t|	[95% conf.	interval]
epds17	3.047074	1.491402	2.04	0.041	.1227528	5.971396
Baseline_psoscore	-.0125248	.0200515	-0.62	0.532	-.0518416	.026792
Baseline_page	.447355	.0573857	7.80	0.000	.3348339	.5598761
Baseline_redu	-.0156446	.2028025	-0.08	0.939	-.4132971	.3820078
Baseline_hedu	.2498628	.1949632	1.28	0.200	-.1324185	.6321441
Baseline_pses	-.5360105	.3681779	-1.46	0.146	-1.257929	.185908
Baseline_reli	1.074789	.2257495	4.76	0.000	.6321418	1.517435
Baseline_par	-.4709472	.3509908	-1.34	0.180	-1.159165	.2172711
Baseline_fdm	2.235939	.664524	3.36	0.001	.9329488	3.538929
Baseline_mdm	2.570961	.5994358	4.29	0.000	1.395595	3.746327
_cons	70.24622	1.566335	44.85	0.000	67.17497	73.31747

Table 15.EPDS cut off score 18 and its correlation with fasting blood sugar

Baseline_prifbs1	Coefficient	Std. err.	t	P>|t|	[95% conf.	interval]
epds18	-.2052862	1.876189	-0.11	0.913	-3.884093	3.473521
Baseline_psoscore	-.0152837	.0200571	-0.76	0.446	-.0546115	.024044
Baseline_page	.4444703	.057414	7.74	0.000	.3318936	.557047
Baseline_redu	-.0150125	.2029526	-0.07	0.941	-.4129594	.3829344
Baseline_hedu	.2431146	.1950767	1.25	0.213	-.1393893	.6256185
Baseline_pses	-.5458733	.3684689	-1.48	0.139	-1.268362	.1766159
Baseline_reli	1.101179	.225958	4.87	0.000	.6581235	1.544235
Baseline_par	-.4570729	.3513597	-1.30	0.193	-1.146015	.2318688
Baseline_fdm	2.217553	.6649513	3.33	0.001	.9137255	3.521381
Baseline_mdm	2.573297	.5998696	4.29	0.000	1.39708	3.749513
_cons	70.44397	1.565545	45.00	0.000	67.37427	73.51367

Table 16-31: Linear regression analysis showing EPDS cut-off score of 3 and above and its correlation with postprandial blood sugar level.

Table 16.EPDS cut off score 3 and its correlation with postprandial blood sugar

Baseline_prippbs1	Coefficient	Std. err.	t	P>|t|	[95% conf.	interval]
epds3	2.412727	1.003756	2.40	0.016	.44454	4.380915
Baseline_psoscore	-.0130267	.0471198	-0.28	0.782	-.1054202	.0793668
Baseline_page	1.655413	.1341009	12.34	0.000	1.392465	1.918361
Baseline_redu	.2390159	.4756149	0.50	0.615	-.6935803	1.171612
Baseline_hedu	-.1917955	.457228	-0.42	0.675	-1.088338	.7047472
Baseline_pses	-.93845	.8701587	-1.08	0.281	-2.644676	.7677763
Baseline_reli	.0333782	.5300797	0.06	0.950	-1.006014	1.07277
Baseline_par	-3.937148	.8247361	-4.77	0.000	-5.554309	-2.319987
Baseline_fdm	4.783966	1.554577	3.08	0.002	1.735717	7.832214
Baseline_mdm	8.843324	1.408381	6.28	0.000	6.08174	11.60491
_cons	70.10933	3.698462	18.96	0.000	62.85731	77.36136

Table 17.EPDS cut off score 4 and its correlation with postprandial blood sugar

Baseline_prippbs1	Coefficient	Std. err.	t	P>|t|	[95% conf.	interval]
epds4	1.896736	1.025101	1.85	0.064	-.1133047	3.906777
Baseline_psoscore	-.0149722	.04717	-0.32	0.751	-.1074641	.0775197
Baseline_page	1.659266	.1341391	12.37	0.000	1.396243	1.922289
Baseline_redu	.238883	.4758219	0.50	0.616	-.694119	1.171885
Baseline_hedu	-.1934113	.4574797	-0.42	0.672	-1.090448	.7036249
Baseline_pses	-1.000567	.8696254	-1.15	0.250	-2.705747	.7046139
Baseline_reli	.0551054	.5301893	0.10	0.917	-.9845014	1.094712
Baseline_par	-3.944431	.8250788	-4.78	0.000	-5.562263	-2.326598
Baseline_fdm	4.900725	1.556152	3.15	0.002	1.849388	7.952062
Baseline_mdm	8.788147	1.408871	6.24	0.000	6.025603	11.55069
_cons	70.47454	3.695685	19.07	0.000	63.22796	77.72112

Table 18.EPDS cut off score 5 and its correlation with postprandial blood sugar

Baseline_prippbs1	Coefficient	Std. err.	t	P>|t|	[95% conf.	interval]
epds5	1.235125	1.051795	1.17	0.240	-.8272572	3.297508
Baseline_psoscore	-.0185247	.0471633	-0.39	0.695	-.1110035	.073954
Baseline_page	1.660594	.1341855	12.38	0.000	1.39748	1.923708
Baseline_redu	.2332866	.4759855	0.49	0.624	-.7000361	1.166609
Baseline_hedu	-.1928253	.4578	-0.42	0.674	-1.09049	.704839
Baseline_pses	-1.030838	.8707147	-1.18	0.237	-2.738155	.6764784
Baseline_reli	.0785431	.5302646	0.15	0.882	-.9612113	1.118298
Baseline_par	-3.950515	.8253958	-4.79	0.000	-5.568969	-2.33206
Baseline_fdm	4.867879	1.556833	3.13	0.002	1.815207	7.920552
Baseline_mdm	8.778689	1.409485	6.23	0.000	6.01494	11.54244
_cons	70.87669	3.692088	19.20	0.000	63.63716	78.11622

Table 19.EPDS cut off score 6 and its correlation with postprandial blood sugar

Baseline_prippbs1	Coefficient	Std. err.	t	P>|t|	[95% conf.	interval]
epds6	1.255542	1.110385	1.13	0.258	-.9217254	3.432809
Baseline_psoscore	-.0177766	.0472533	-0.38	0.707	-.110432	.0748788
Baseline_page	1.661104	.1341859	12.38	0.000	1.397989	1.924219
Baseline_redu	.2265852	.4760219	0.48	0.634	-.7068089	1.159979
Baseline_hedu	-.1948951	.4578974	-0.43	0.670	-1.09275	.7029603
Baseline_pses	-1.027992	.8711848	-1.18	0.238	-2.736231	.6802461
Baseline_reli	.0792955	.5302987	0.15	0.881	-.9605257	1.119117
Baseline_par	-3.962894	.825331	-4.80	0.000	-5.581221	-2.344567
Baseline_fdm	4.845099	1.556309	3.11	0.002	1.793454	7.896744
Baseline_mdm	8.7638	1.409748	6.22	0.000	5.999536	11.52806
_cons	70.94718	3.68658	19.24	0.000	63.71846	78.17591

Table 20.EPDS cut off score 7 and its correlation with postprandial blood sugar

Baseline_prippbs1	Coefficient	Std. err.	t	P>|t|	[95% conf.	interval]
epds7	.722235	1.158579	0.62	0.533	-1.549533	2.994003
Baseline_psoscore	-.0206688	.0472369	-0.44	0.662	-.113292	.0719543
Baseline_page	1.661798	.1342073	12.38	0.000	1.398641	1.924954
Baseline_redu	.2281602	.4761199	0.48	0.632	-.7054262	1.161747
Baseline_hedu	-.1849355	.457921	-0.40	0.686	-1.082837	.7129661
Baseline_pses	-1.061009	.8722718	-1.22	0.224	-2.771379	.6493606
Baseline_reli	.0917858	.530608	0.17	0.863	-.948642	1.132214
Baseline_par	-3.961651	.825472	-4.80	0.000	-5.580255	-2.343048
Baseline_fdm	4.824347	1.556519	3.10	0.002	1.77229	7.876403
Baseline_mdm	8.781483	1.409911	6.23	0.000	6.016899	11.54607
_cons	71.17739	3.687294	19.30	0.000	63.94727	78.40752

Table 21.EPDS cut off score 8 and its correlation with postprandial blood sugar

Baseline_prippbs1	Coefficient	Std. err.	t	P>|t|	[95% conf.	interval]
epds8	.4923536	1.217262	0.40	0.686	-1.894481	2.879188
Baseline_psoscore	-.0217619	.047253	-0.46	0.645	-.1144166	.0708929
Baseline_page	1.661966	.1342157	12.38	0.000	1.398793	1.925139
Baseline_redu	.2295394	.476144	0.48	0.630	-.7040942	1.163173
Baseline_hedu	-.1807677	.457895	-0.39	0.693	-1.078618	.717083
Baseline_pses	-1.083165	.8715907	-1.24	0.214	-2.792199	.6258693
Baseline_reli	.0980764	.5307826	0.18	0.853	-.9426938	1.138847
Baseline_par	-3.965494	.8255044	-4.80	0.000	-5.584161	-2.346827
Baseline_fdm	4.817116	1.556638	3.09	0.002	1.764827	7.869406
Baseline_mdm	8.79615	1.409706	6.24	0.000	6.031968	11.56033
_cons	71.28561	3.684939	19.35	0.000	64.0601	78.51112

Table 22.EPDS cut off score 9 and its correlation with postprandial blood sugar

Baseline_prippbs1	Coefficient	Std. err.	t	P>|t|	[95% conf.	interval]
epds9	.9364358	1.301358	0.72	0.472	-1.615295	3.488167
Baseline_psoscore	-.0203221	.0472123	-0.43	0.667	-.112897	.0722528
Baseline_page	1.663021	.1342187	12.39	0.000	1.399842	1.9262
Baseline_redu	.222344	.4762663	0.47	0.641	-.7115294	1.156217
Baseline_hedu	-.1859242	.4578799	-0.41	0.685	-1.083745	.7118967
Baseline_pses	-1.05769	.8715708	-1.21	0.225	-2.766686	.6513047
Baseline_reli	.0856833	.530864	0.16	0.872	-.9552465	1.126613
Baseline_par	-3.968596	.825468	-4.81	0.000	-5.587192	-2.35
Baseline_fdm	4.820507	1.55627	3.10	0.002	1.768938	7.872076
Baseline_mdm	8.779304	1.409863	6.23	0.000	6.014813	11.5438
_cons	71.17687	3.680243	19.34	0.000	63.96056	78.39317

Table 23.EPDS cut off score 10 and its correlation with postprandial blood sugar

Baseline_prippbs1	Coefficient	Std. err.	t	P>|t|	[95% conf.	interval]
epds10	1.286853	1.417089	0.91	0.364	-1.491806	4.065512
Baseline_psoscore	-.0192714	.0472222	-0.41	0.683	-.1118658	.073323
Baseline_page	1.662621	.1342012	12.39	0.000	1.399476	1.925766
Baseline_redu	.2245964	.4761112	0.47	0.637	-.708973	1.158166
Baseline_hedu	-.1910993	.4579443	-0.42	0.676	-1.089046	.7068479
Baseline_pses	-1.040186	.8717661	-1.19	0.233	-2.749564	.6691923
Baseline_reli	.0772088	.5309503	0.15	0.884	-.9638903	1.118308
Baseline_par	-3.964296	.8253981	-4.80	0.000	-5.582755	-2.345838
Baseline_fdm	4.82468	1.556167	3.10	0.002	1.773313	7.876047
Baseline_mdm	8.768395	1.409922	6.22	0.000	6.003788	11.533
_cons	71.12922	3.676964	19.34	0.000	63.91934	78.33909

Table 24.EPDS cut off score 11 and its correlation with postprandial blood sugar

Baseline_prippbs1	Coefficient	Std. err.	t	P>|t|	[95% conf.	interval]
epds11	-.2836421	1.545725	-0.18	0.854	-3.314534	2.74725
Baseline_psoscore	-.0248673	.047228	-0.53	0.599	-.1174731	.0677384
Baseline_page	1.661732	.1342181	12.38	0.000	1.398554	1.92491
Baseline_redu	.2327992	.4761044	0.49	0.625	-.7007567	1.166355
Baseline_hedu	-.1723728	.457797	-0.38	0.707	-1.070031	.7252856
Baseline_pses	-1.127337	.8703253	-1.30	0.195	-2.83389	.5792159
Baseline_reli	.1198028	.5311298	0.23	0.822	-.9216482	1.161254
Baseline_par	-3.965011	.8255327	-4.80	0.000	-5.583734	-2.346288
Baseline_fdm	4.794865	1.556551	3.08	0.002	1.742746	7.846984
Baseline_mdm	8.804272	1.410015	6.24	0.000	6.039483	11.56906
_cons	71.51823	3.672142	19.48	0.000	64.31781	78.71865

Table 25.EPDS cut off score 12 and its correlation with postprandial blood sugar

Baseline_prippbs1	Coefficient	Std. err.	t	P>|t|	[95% conf.	interval]
epds12	.7666657	1.714922	0.45	0.655	-2.595991	4.129323
Baseline_psoscore	-.0214557	.0472729	-0.45	0.650	-.1141493	.071238
Baseline_page	1.661657	.1342116	12.38	0.000	1.398492	1.924822
Baseline_redu	.2340135	.4760991	0.49	0.623	-.6995322	1.167559
Baseline_hedu	-.1763383	.4576502	-0.39	0.700	-1.073709	.7210322
Baseline_pses	-1.095186	.8690988	-1.26	0.208	-2.799334	.6089618
Baseline_reli	.0955818	.5309308	0.18	0.857	-.945479	1.136643
Baseline_par	-3.962038	.8255035	-4.80	0.000	-5.580703	-2.343372
Baseline_fdm	4.827026	1.557203	3.10	0.002	1.773628	7.880424
Baseline_mdm	8.792331	1.409758	6.24	0.000	6.028048	11.55662
_cons	71.31229	3.674337	19.41	0.000	64.10757	78.51701

Table 26.EPDS cut off score 13 and its correlation with postprandial blood sugar

Baseline_prippbs1	Coefficient	Std. err.	t	P>|t|	[95% conf.	interval]
Epds13	1.405024	1.928893	0.73	0.466	-2.377191	5.187239
Baseline_psoscore	-.0209026	.0471289	-0.44	0.657	-.1133139	.0715087
Baseline_page	1.661916	.1342042	12.38	0.000	1.398766	1.925067
Baseline_redu	.230353	.4760666	0.48	0.629	-.7031289	1.163835
Baseline_hedu	-.1790688	.4576474	-0.39	0.696	-1.076434	.7182962
Baseline_pses	-1.086919	.8687443	-1.25	0.211	-2.790372	.616534
Baseline_reli	.0853457	.5308637	0.16	0.872	-.9555836	1.126275
Baseline_par	-3.963254	.8254426	-4.80	0.000	-5.5818	-2.344708
Baseline_fdm	4.834754	1.556718	3.11	0.002	1.782308	7.887201
Baseline_mdm	8.785561	1.409715	6.23	0.000	6.02136	11.54976
_cons	71.28114	3.667271	19.44	0.000	64.09028	78.47201

Table 27.EPDS cut off score 14 and its correlation with postprandial blood sugar

Baseline_prippbs1	Coefficient	Std. err.	t	P>|t|	[95% conf.	interval]
epds14	3.175274	2.189207	1.45	0.147	-1.117372	7.467919
Baseline_psoscore	-.0187818	.0470661	-0.40	0.690	-.11107	.0735064
Baseline_page	1.663144	.1341696	12.40	0.000	1.400061	1.926227
Baseline_redu	.2389174	.4759435	0.50	0.616	-.694323	1.172158
Baseline_hedu	-.1803492	.4574925	-0.39	0.693	-1.07741	.7167122
Baseline_pses	-1.080311	.8679498	-1.24	0.213	-2.782206	.6215843
Baseline_reli	.0692352	.5302464	0.13	0.896	-.9704837	1.108954
Baseline_par	-3.981826	.8252983	-4.82	0.000	-5.600089	-2.363563
Baseline_fdm	4.86242	1.556165	3.12	0.002	1.811057	7.913783
Baseline_mdm	8.797173	1.409195	6.24	0.000	6.033993	11.56035
_cons	71.12051	3.665399	19.40	0.000	63.93332	78.30771

Table 28.EPDS cut off score 15 and its correlation with postprandial blood sugar

Baseline_prippbs1	Coefficient	Std. err.	t	P>|t|	[95% conf.	interval]
epds15	3.234189	2.455827	1.32	0.188	-1.58125	8.049628
Baseline_psoscore	-.0185621	.0471118	-0.39	0.694	-.1109399	.0738158
Baseline_page	1.663935	.1341862	12.40	0.000	1.40082	1.92705
Baseline_redu	.2363882	.4759637	0.50	0.619	-.696892	1.169668
Baseline_hedu	-.1769074	.457509	-0.39	0.699	-1.074001	.7201862
Baseline_pses	-1.081054	.8680628	-1.25	0.213	-2.78317	.6210628
Baseline_reli	.0785227	.5300705	0.15	0.882	-.9608512	1.117897
Baseline_par	-3.981523	.825369	-4.82	0.000	-5.599925	-2.363121
Baseline_fdm	4.867362	1.556508	3.13	0.002	1.815327	7.919397
Baseline_mdm	8.785288	1.409327	6.23	0.000	6.021849	11.54873
_cons	71.12196	3.667173	19.39	0.000	63.93129	78.31263

Table 29.EPDS cut off score 16 and its correlation with postprandial blood sugar

Baseline_prippbs1	Coefficient	Std. err.	t	P>|t|	[95% conf.	interval]
epds16	3.023588	2.993996	1.01	0.313	-2.847106	8.894282
Baseline_psoscore	-.0207685	.0470455	-0.44	0.659	-.1130163	.0714793
Baseline_page	1.664298	.1342186	12.40	0.000	1.40112	1.927477
Baseline_redu	.2287935	.4760296	0.48	0.631	-.7046158	1.162203
Baseline_hedu	-.1720766	.4575709	-0.38	0.707	-1.069292	.7251385
Baseline_pses	-1.092239	.8680863	-1.26	0.208	-2.794401	.6099242
Baseline_reli	.0840954	.5302559	0.16	0.874	-.9556421	1.123833
Baseline_par	-3.973706	.8254243	-4.81	0.000	-5.592216	-2.355196
Baseline_fdm	4.846577	1.556547	3.11	0.002	1.794467	7.898688
Baseline_mdm	8.785239	1.409536	6.23	0.000	6.02139	11.54909
_cons	71.23625	3.665364	19.43	0.000	64.04912	78.42338

Table 30.EPDS cut off score 17 and its correlation with postprandial blood sugar

Baseline_prippbs1	Coefficient	Std. err.	t	P>|t|	[95% conf.	interval]
epds17	2.314059	3.430738	0.67	0.500	-4.413006	9.041125
Baseline_psoscore	-.0218563	.0470465	-0.46	0.642	-.1141061	.0703935
Baseline_page	1.663894	.1342491	12.39	0.000	1.400655	1.927132
Baseline_redu	.2322124	.4760645	0.49	0.626	-.7012654	1.16569
Baseline_hedu	-.1698567	.4576636	-0.37	0.711	-1.067254	.7275403
Baseline_pses	-1.107667	.8679469	-1.28	0.202	-2.809556	.5942225
Baseline_reli	.0922049	.5304154	0.17	0.862	-.9478453	1.132255
Baseline_par	-3.973163	.8255638	-4.81	0.000	-5.591947	-2.35438
Baseline_fdm	4.816203	1.556215	3.09	0.002	1.764742	7.867665
Baseline_mdm	8.797085	1.409617	6.24	0.000	6.033077	11.56109
_cons	71.308	3.666022	19.45	0.000	64.11959	78.49642

Table 31.EPDS cut off score 18 and its correlation with postprandial blood sugar

Baseline_prippbs1	Coefficient	Std. err.	t	P>|t|	[95% conf.	interval]
epds18	**3.55084**	**4.311581**	**0.82**	**0.410**	**-4.903404**	**12.00508**
Baseline_psoscore	**-.0216872**	**.047023**	**-0.46**	**0.645**	**-.1138909**	**.0705166**
Baseline_page	**1.662943**	**.1342101**	**12.39**	**0.000**	**1.399781**	**1.926105**
Baseline_redu	**.2300863**	**.4760539**	**0.48**	**0.629**	**-.7033706**	**1.163543**
Baseline_hedu	**-.1743445**	**.4575928**	**-0.38**	**0.703**	**-1.071603**	**.7229135**
Baseline_pses	**-1.102628**	**.8679749**	**-1.27**	**0.204**	**-2.804572**	**.5993165**
Baseline_reli	**.086295**	**.5304958**	**0.16**	**0.871**	**-.9539129**	**1.126503**
Baseline_par	**-3.984839**	**.8258059**	**-4.83**	**0.000**	**-5.604098**	**-2.365581**
Baseline_fdm	**4.807828**	**1.556014**	**3.09**	**0.002**	**1.756762**	**7.858894**
Baseline_mdm	**8.79559**	**1.409563**	**6.24**	**0.000**	**6.031688**	**11.55949**
_cons	**71.35477**	**3.661088**	**19.49**	**0.000**	**64.17603**	**78.53352**
